# SFEM Analysis of Beams with Scaled Lengths including Spatially Varying and Cross-Correlated Concrete Properties

**DOI:** 10.3390/ma15010095

**Published:** 2021-12-23

**Authors:** Ewelina Korol

**Affiliations:** Faculty of Civil and Environmental Engineering, Gdansk University of Technology, Narutowicza 11/12, 80-233 Gdansk, Poland; ewelina.korol@pg.edu.pl

**Keywords:** concrete beam, coefficient of variation, cross-correlation, elasto-plasticity, non-local softening, random field, SFEM, statistical size effect

## Abstract

This paper presents the results obtained for plain concrete beams under four-point bending with spatially varying material properties. Beams of increasing length but constant depth were analyzed using the stochastic finite element method. Spatial fluctuation of a uniaxial tensile strength, fracture energy and elastic modulus was defined within cross-correlated random fields. The symmetrical Gauss probability distribution function was applied for the material properties. The shape of the probability distribution function was modified by changing the coefficient of variation in order to find its right value. The correctness of the numerical solution was verified against the experimental results of Koide et al. (1998, 2000). The stochastic FEM analysis was performed with an autocorrelation length of 40 mm and material coefficients of variation of 0.12, 0.14, 0.16, 0.20 and 0.24. The comparison between numerical outcomes and experimental results demonstrated that the coefficient of variation of 0.24 gave the best agreement when referring to the experimental mean values. On the other hand, the variation of results was better captured with the coefficient of variation of 0.16. The findings indicate that the Gauss probability distribution function with *cov* = 0.24 correctly reproduced the statistical size effect, but its tails needed modification in order to project experimental result variation.

## 1. Introduction

The size effect in quasi-brittle materials has been commonly identified as a nominal strength reduction connected with an increasing specimen size. This statement is particularly valid for geometrically similar structures (i.e., when a specimen is scaled in the directions of the length *L* and depth *D* simultaneously). The requirement of geometrical similarity provides the same failure mechanism. There are two main mechanical sources of the size effect in concrete: energetic and statistical ones. The energetic size effect is connected with a strain localization phenomenon that develops before the macro-crack appears. The fracture process zone size (or strain localization zone) is a material property that is dependent mostly on the material characteristic length of a microstructure *l_c_*. When the beam depth *D* increases (i.e., *l_c_*/*D*→0), the nominal strength decreases together with its ductility. The energetic size effect is of a deterministic nature, and it assumes a homogenous (uniform) distribution of the material properties. On the other hand, the statistical size effect is caused by a spatial fluctuation of the material properties. Generally, the number of flaws inside the material increases with an increasing specimen size. Hence, the strength reduction becomes stronger with an increasing specimen size. The weakest link theory introduced by Weibull [1] postulates that a structure is as strong as its weakest component. Thus, the structure fails when its strength is exceeded in the weakest spot, since stress redistribution is not considered. The Weibull theory is valid for large structures, which fail as soon as a macroscopic fracture initiates in one small material element. However, it is not able to account for a spatial correlation of the local material properties and does not include the material characteristic length *l_c_* (i.e., it ignores the energetic size effect). As a result, the weakest link theory underestimates the strength of small- and medium-sized specimens.

The combination of the energetic and statistical size effects was introduced by Bazant [2] into the analytical type I size effect law. The coupled energetic-statistical size effect covers the whole size range from small to large specimens. The nominal strength described by the type I SEL applies to geometrically similar structures of positive geometries without initial notches or large-stress free cracks. The last condition is crucial for the statistical part of the size effect, since it enables the FPZ to develop at a random position. When the position of the FPZ is fixed (e.g., by a notch), the statistical size effect on the mean nominal strength is negligible. In that case, the nominal strength can be described by the type II SEL from Bazant and Planas [3], which represents the energetic size effect solely. Reinforced concrete beams failing due to concrete damage follows the type II SEL. It was numerically demonstrated by Syroka-Korol et al. [4] that when referring to RC beams experiencing shear failure, the material randomness only causes spreading of the results.

The coupled energetic-statistical size effect is commonly verified against experimental research with plain concrete beams [5] or dog bone specimens [6]. The isolated statistical size effect can be analyzed with plain concrete beams of varying lengths but a constant depth [7,8]. Numerical analysis of statistical sources of the size effect requires the application of concrete parameter spatial fluctuation, which is mostly performed within autocorrelated random fields. The type of autocorrelation function and autocorrelation length have influence on the parameters’ variation in space. Generally, the square exponential autocorrelation function is adopted [4]. The probability distribution of concrete’s material properties was described by Weibull [9], grafted Weibull–Gauss [10] and Gauss [11] probability distribution functions (pdfs). The shape of the pdf was modified by changing the shape parameter *m* or the coefficient of variation *cov* in the Weibull and Gauss functions, respectively.

The comprehensive numerical investigation of a pure statistical size effect in plain concrete beams subjected to four-point bending was performed by, among others, Bazant and Novak [9] and also by Grassl and Bazant [10]. Bazant and Novak [9] performed simulations using a stochastic finite element method and the Weibull pdf to describe the concrete’s parameter variations. The experimental mean bending moments registered by Koide et al. [7,8] were numerically covered with a surprisingly low Weibull modulus of *m* = 8. Next, Grassl and Bazant [10] analyzed the same beams with the aid of a lattice model. It was concluded that the geometrical randomness in the lattice model was insufficient to reproduce the experimentally observed average strength reduction from Koide et al. [7,8]. The fluctuation of the lattice bars’ strength was modeled with autocorrelated random fields with a square exponential autocorrelation function and the autocorrelation length *l_cor_* = 40 mm. The tensile strength *f_t_* variability was indirectly defined through *ε_0_* (strain corresponding to *f_t_*), and its coefficient of variation was *cov* = 0.25. This time, the grafted Gaussian–Weibull cumulative distribution function was adopted. (It was demonstrated in [12,13] that the tails below a probability of 0.01 followed Weibull, while the core was Gaussian.) The numerical results obtained for the longest and medium beams exhibited good agreement with the experimental findings. However, the shortest beam overestimated the experimental result variations.

The combined energetic-statistical size effect was investigated within a stochastic discrete meso-scale model by Elias et al. [14]. Simulations of geometrically similar concrete beams subjected to three-point bending of a depth *D* = 40, 93, 215 and 500 mm were performed. A relative notch length varied between *α_0_* = 0 ÷ 0.3D (the statistical size effect appeared in unnotched beams with *α_0_* = 0 only). A lattice–particle model was used to simulate concrete fractures. The spatially autocorrelated random fields were introduced to define the material properties. As a result, the model considered both the geometrical and material randomness. The grafted Weibull–Gaussian probability distribution function was adopted. As suggested in [10], the material coefficient of variation was *cov* = 0.25, while the autocorrelation length *l_cor_* was 40 and 80 mm. The stochastic results (with both *l_cor_*) obtained for the two largest beam sizes (*D* = 215 and 500 mm) highly underestimated the experimentally observed average nominal strength (experiments performed by Hoover et al. [15]). In other words, the simulated statistical size effect with *cov* = 0.25 was too strong compared with the laboratory tests. Satisfactory agreement was achieved only for small beams with *D* = 40 and 93 mm, where the statistical size effect was relatively weak and the energetic size effect dominated.

The finite element method was applied by Syroka-Korol et al. [11] to also investigate the combined energetic-statistical size effect in plain concrete beams under three-point bending. The very broad range of the autocorrelation length *l_cor_* = 5, 10, 15, 20, 40, 60, 80, 100, 120, 140 and 150 mm was analyzed in geometrically similar beams of depth *D* = 80, 160, 320 and 1920 mm. Concrete was modeled within elaso-plasticity with non-local softening. The fluctuation of the local material strength was described by the square exponential autocorrelation function, and the pdf was entirely Gaussian. The results showed a strong effect of the autocorrelation length *l_cor_* upon the average nominal strength reduction. It was demonstrated that the nominal strength depends on the FPZ size at the peak load, the average local material strength inside the FPZ and its position with respect to the support. This extensive SFEM study showed that the lowest average nominal strength in all beam sizes was obtained with the autocorrelation length *l_cor_* = 0.5*h_loc_* (*h_loc_* = the height of the FPZ at the peak load), while the strongest reduction of the average nominal strength with increasing beam depth *D* (i.e., the strongest statistical size effect) was obtained for *l_cor_* = 40 mm.

The purpose of the current study is to calibrate the stochastic parameters of the FE model to properly describe the pure statistical size effect. Plain concrete beams of series C tested by Koide et al. [7,8] are the comparative base. The stochastic finite element method is chosen because, in contrast to the lattice model [10] and lattice–particle model [14], it is applicable to both small and very large specimens (compare SFEM analysis of 6-m long RC beams in [4]) and can be further used to analyze the safety of real-sized structures. The fluctuation of the material parameters defined within random fields is described by the square exponential autocorrelation function and the autocorrelation length *l_cor_* = 40 mm, as already validated in [11]. The entirely Gaussian pdf is applied, which is a simplification compared with [10,14], where the core is Gaussian but the tails follow the Weibull pdf. The effect of varying the material coefficient of variation *cov* is carefully studied. The SFEM simulations were performed with simultaneously varying cross-correlated random distributions of the local uniaxial tensile strength, fracture energy and elastic modulus in contrast to [14], where the material parameters are mutually linearly dependent.

The outline of the present paper is as follows. First, after the introduction (Section 1), the numerical SFEM model is presented in Section 2. The numerical results from SFEM simulations are discussed in Section 3. The conclusions are listed in Section 4.

## 2. Materials and Methods

### 2.1. Constitutive Elasto-Plastic Model with Non-Local Softening

**Figure 1:**
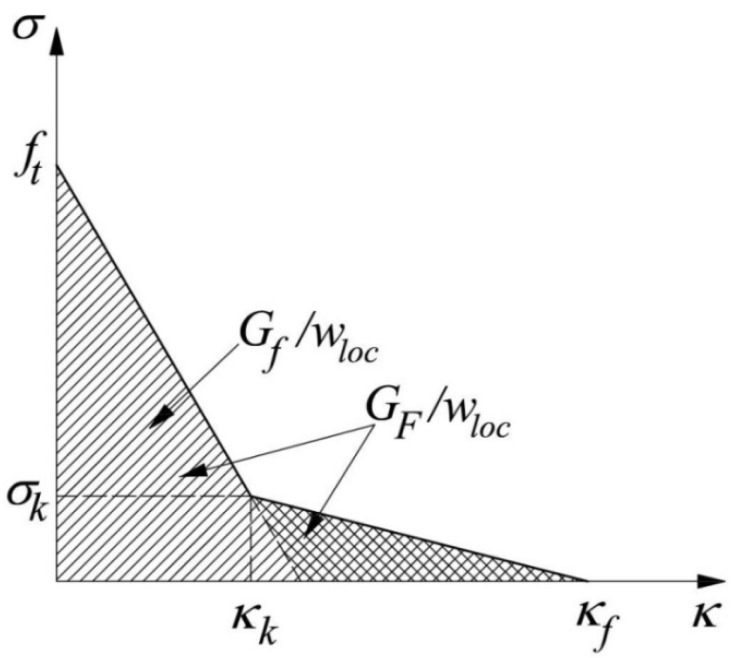
The bilinear softening function under tension expressed in terms of tensile stress *σ_t_* versus the softening parameter *κ* (*G_f_* = initial fracture energy, *G_F_* = total fracture energy and *w_loc_* = the localized zone width).

The concrete behavior under tension was defined with a simple Rankine criterion with isotropic softening, while the yield function *f* was as follows:

(1) $$f=max\left\{\sigma_{1},\sigma_{2},\sigma_{3}\right\}-\sigma_{t}\left(\kappa \right),$$

where *σ_i_*, *i* = 1, 2, 3 are the principal stresses, *σ_t_* is the tensile yield stress and *κ* is the softening parameter equal to the maximum principal plastic strain *ε_1p_* [11]. The associated flow rule was assumed. The bilinear function recommended by Hoover and Bazant [15] (Figure 1) was used to describe the post-peak softening under tension. The total fracture energy *G_F_* = 80 N/m was calculated after the CEB-FIP code [16], based on the experimentally measured concrete compressive strength *f_c_* = 30 MPa and the maximum aggregate diameter *d_a_* = 20 mm. The ratio between the total and initial fracture energy was taken to be *G_F_*/*G_f_* = 1.5 (very close to recommendation in [15]). The uniaxial tensile strength *f_t_* was not experimentally measured by Koide et al. [7,8]. Nevertheless, *f_t_* = 2.9 MPa was initially adopted (estimated based on *f_c_* and the recommendations in Eurocode 2 [17]).

In the calculations, a non-local regularization technique was applied, and a non-local formulation of the softening parameter was introduced [11]:

(2) $$\bar{\kappa }\left(x\right)=\left(1-m_{nl}\right)\kappa \left(x\right)+m_{nl}\frac{\int_{V}\omega \left(\left\|x-\xi \right\|\right)\kappa \left(\xi \right)d\xi }{\int_{V}\omega \left(\left\|x-\xi \right\|\right)d\xi },\;with\;i=1,\;2,$$

where *V* is the body volume, ***x*** is the coordinate vector of the analyzed point, *ξ* is the coordinate vector of the surrounding points and *m_nl_* is the non-local parameter. The normal distribution function was used as a weighting function:

(3) $$\omega \left(r\right)=\frac{1}{l_{c}\sqrt{\pi }}e^{-{\left(\frac{r}{l_{c}}\right)}^{2}}$$

This depended on the characteristic length of the microstructure *l_c_* and the distance between material points *r*. The characteristic length was assumed in this study to be *l_c_* = 2 mm, based on the experimental investigations by Skarzynski and Tejchman [18]. The softening non-local parameters near the boundaries were always calculated on the basis of Equations (2) and (3), which satisfied the normalizing condition.

Calculations were performed in the commercial finite element software ABAQUS from Dassault Systèmes Simulia Corp. (Rhode Island, US) with user subroutines which introduced the non-local model, constitutive law and user element definition. To capture the whole load-deflection curves, including the post-peak softening, the arc-length method was used to control the load (the simplified procedure formulated by Jirasek and Bazant [19]).

### 2.2. Random Fields

The local material properties had the Gauss probability distribution function with a prescribed mean value *μ* and a standard deviation *sdev*. The coefficient of variation was defined by the ratio *cov* = *sdev/μ* (the tensile strength, elastic modulus and fracture energy had the same coefficient of variation, calculated as *cov_ft_* = σ_ft_/*μ_ft_*, *cov_Emod_* = σ_Emod_/*μ_Emod_* and *cov_Gf_* = σ_Gf_/*μ_Gf_*, respectively). The material fluctuation was represented by spatially autocorrelated random fields with a homogenous squared exponential autocorrelation function:

(4) $$C\left(x_{1},x_{2}\right)=exp\left(\frac{-{\left(x_{1}-x_{2}\right)}^{2}}{l_{cor}^{2}}\right)$$

where |*x*_1_ − *x*_2_| is the distance between two points in a Cartesian space and *l_cor_* is the autocorrelation length, assumed to be *l_cor_* = 40 mm. The auto-covariance function had the following spectral decomposition:

(5) $$C\left(x_{1},x_{2}\right)=\overset{\infty }{\underset{i=1}{\sum }}\lambda_{i}f_{i}\left(x_{1}\right)f_{i}\left(x_{2}\right)$$

where the eigenvalues *λ_i_* and eigenfunctions *f_i_*(***x***) are the solution of the Fredholm integral equation of the second kind:

(6) $$\int_{D}C\left(x_{1},x_{2}\right)f_{i}\left(x_{1}\right)dx_{1}=\lambda_{i}f_{i}\left(x_{2}\right)$$

The solution to Equation (6) was found with the aid of the wavelet-Galerkin method described by Phoon et al. [20,21], where Haar wavelets are implemented to create an orthogonal base.

The spatially correlated random fields *H*(***x***,*θ*) (here with the zero mean and unit variance) were defined according to the Karhunen–Loëve expansion [22,23] by an infinite linear combination of orthogonal functions and random coefficients:

(7) $$H\left(x,\theta \right)=\overset{\infty }{\underset{i=1}{\sum }}\sqrt{\lambda_{i}}\xi_{i}\left(\theta \right)f_{i}\left(x\right)$$

where *ξ_i_*(*θ*) is the vector of uncorrelated random variables sampled from N(0,1) distribution, *λ*_i_ is the eigenvalues and *f_i_*(***x***) is the corresponding eigenfunctions. The approximated solution $\;\hat{H}\left(x,\theta \right)$ was obtained by truncating the series in Equation (7) after *M* terms which satisfied the condition $\overset{M}{\underset{i=1}{\sum }}\lambda_{i}0.95tr\left(C\right)$.

**Figure 2:**
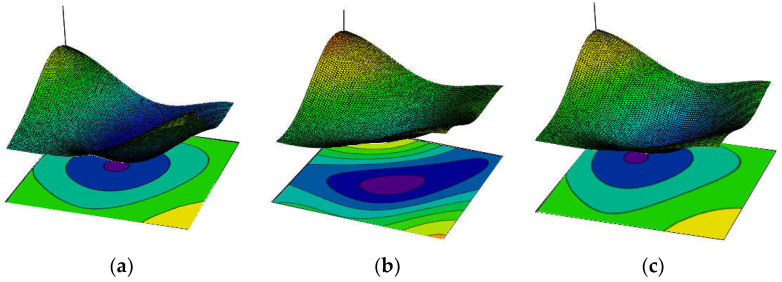
Exemplary profiles of cross-correlated and spatially auto-correlated random distributions of local material parameters: (**a**) *f_t_*, (**b**) *G_f_* and (**c**) *E_mod_*.

The simultaneously varying local uniaxial tensile strength *f_t_*, fracture energy *G_f_* and elasticity modulus *E_mod_* were simply cross-correlated through a scalar coefficient *r* as proposed by Vorechovsky [24]. When the cross-correlation coefficient *r* = 0, the spatial distributions of the material parameters are independent. On the other hand, when *r* = 1, the material parameters are linearly dependent. In the present SFEM study, a strong cross-correlation (*r* = 0.9) was assumed between *f_t_*, *G_f_* and *E* (exemplary profiles are given in Figure 2).

### 2.3. FE Input Data

**Figure 3:**
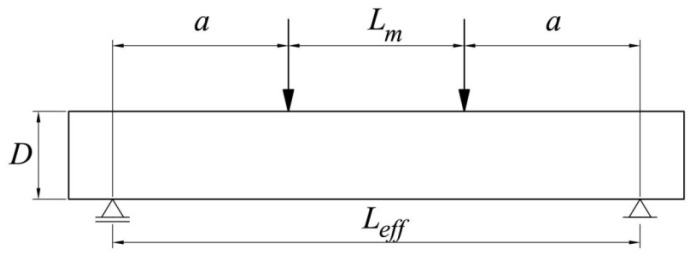
Geometry of analyzed beams.

Three beam sizes of a constant depth *D* = 100 mm and thickness *t* = 100 mm but varying span were analyzed, similar to Series C as tested by Koide et al. [7,8]. The beams were subjected to four-point bending, and the distance from the support to the loading point was always *a* = 200 mm (Figure 3). Hence, the energetic size effect was negligible, and the predominant statistical size effect could be investigated. The beam’s effective span *L_eff_* varied from 600 mm through 800 mm and up to 1000 mm, while the corresponding distance between loading points was *L_m_* = 200, 400 and 600 mm (beams denoted as LM200, LM400 and LM600, respectively). Series C, as tested by Koide et al. [7,8], consisted of 121 plain concrete beams, including 46, 40 and 35 identical specimens of sizes LM200, LM400 and LM600, respectively.

**Figure 4:**
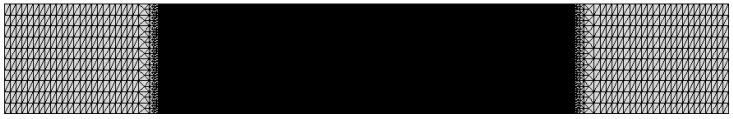
FE mesh of analyzed beam LM200.

A large central part of the beam, where the non-local elasto-plastic material was defined, was covered by a fine FE mesh consisting of right triangles of dimensions 1 mm × 1.25 mm (Figure 4). The remaining part of the beam, which consisted of a coarse mesh, was defined as an elastic material. Analysis was performed under plain stress conditions. The initial calculations started with the following concrete properties: uniaxial tensile strength *f_t_* = 2.9 MPa, initial fracture energy *G_f_* = 52 N/m, total fracture energy *G_F_* = 80 N/m, elasticity modulus *E_mod_* = 30 GPa and Poisson’s ratio *ν* = 0.17. The nominal strength of a beam expressed as *σ_N_* = *M_max_*/(*tD*^2^/6) was linearly dependent on *M_max_* due to the constant cross-section dimensions. Hence, the terms nominal strength and bending moment were used interchangeably when describing the results.

## 3. Results

### 3.1. Convergence Study

**Figure 5:**
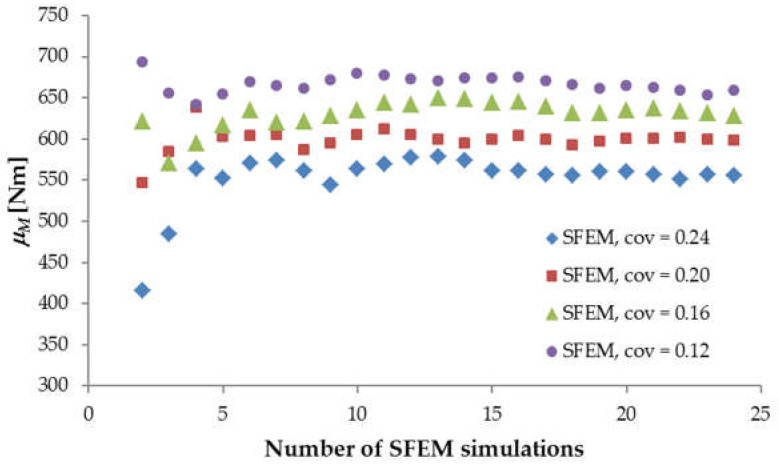
The calculated mean ultimate bending moment *μ_M_* against the number of SFEM simulations for beam LM200.

**Figure 6:**
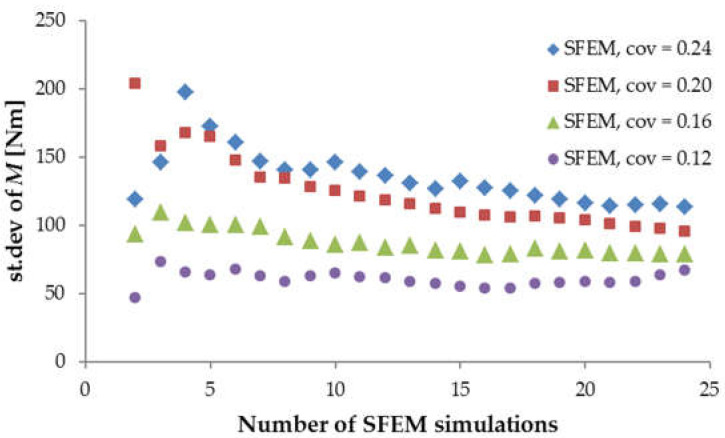
The calculated standard deviation of the ultimate bending moment *M* against the number of SFEM simulations for beam LM200.

Initially, 2000 random fields were generated for each beam size and each material parameter. In order to reduce the computational time, the stratified sampling technique [11] was applied, and as a result, 24 random fields were eventually chosen for further FE simulations. Stochastic analysis started with the shortest beam, denoted as LM200 with simultaneously varying *f_t_*, *G_f_* and *E_mod_*, for which a strong cross-correlation was assumed to be *r* = 0.9. The material coefficients of variation (identical for *f_t_*, *G_f_* and *E_mod_*) changed from *cov* = 0.12 through 0.16 and 0.20 and up to 0.24 (for each *cov*, the new set of random fields was generated). First, convergence analysis was performed in order to verify whether the specified number of SFEM simulations provided a satisfactory approximation of the desired solution. Figure 5 compares the calculated mean values of the ultimate bending moment obtained for an increasing number of SFEM simulations with different material *cov* values. Initially, when the number of simulations was under five, the scattering of the mean value *μ_M_* was very strong, particularly for a higher material *cov*. After 10 simulations, the fluctuation of *μ_M_* weakened, and after ca. 20 simulations, the changes were unnoticeable for all material *cov* values. Referring to the standard deviation of ultimate *M* (Figure 6), its convergence was slightly slower than that observed for the mean value, although after 20 simulations, the obtained standard deviations showed acceptably low changes. Thus, the chosen number of 24 simulations turned out to give adequate results.

The numerical results of beam LM200 presented in Figure 5 and Figure 6 clearly show that the increasing material *cov* caused a reduction in the mean ultimate bending moment *μ_M_* and an increase in its standard deviation. Moreover, the resulting coefficient of variation *COV_M_* (st.dev.*M*/*μ_M_*) was always lower than the assumed material *cov*. This behavior can be explained as follows. The beam’s ultimate strength depends on the value of the local *f_t_* and *G_f_* inside the localized zone (or the FPZ) at the peak load. When the FPZ dimensions were larger than the autocorrelation length, the average value of the varying parameters *f_t_* and *G_f_* inside the FPZ gave lower values than the input material *cov*. Hence, the resulting ultimate failure force and corresponding bending moment had a lower coefficient of variation *COV_M_*.

### 3.2. Material and Stochastic Parameter Calibration

**Figure 7:**
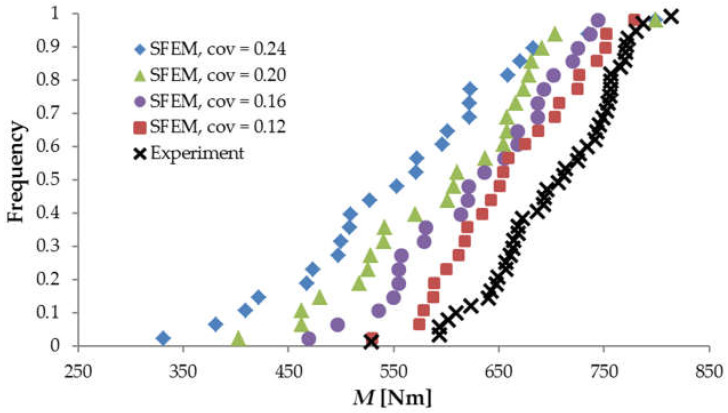
The original frequency diagrams for beam LM200 obtained for different material *cov* compared with the experimental results of Koide et al. (Copyright Permission, Proceedings of FRAMCOS-3, 1998 and Proceedings of ICASP-8, 2000) [7,8].

**Figure 8:**
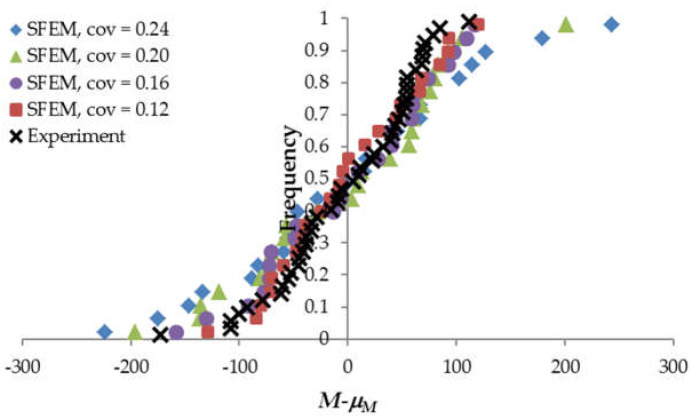
The translated frequency diagrams for beam LM200 obtained for different material *cov* compared with the experimental results of Koide et al. (Copyright Permission, Proceedings of FRAMCOS-3, 1998 and Proceedings of ICASP-8, 2000) [7,8].

Figure 7 compares the experimental and numerical frequency diagrams obtained for beam LM200 with various material *cov* values. The calculated mean ultimate bending moment *μ_M_* reached 659, 627, 598 and 555 Nm for material *cov* values of 0.12, 0.16, 0.20 and 0.24, respectively, while the corresponding standard deviation of *M* was 67, 78, 95 and 113 Nm. It is clear that the calculated mean ultimate bending moment did not coincide with the experimental one. However, the effect of the varying material *cov* upon the resulting variation of *M* could be still verified against the experimental outcomes based on the translated frequency distributions (Figure 8) (translated by the calculated mean value *μ_M_* for each distribution separately). The experimental frequency distribution was subdivided into 24 strata (the same number as in the SFEM calculations), keeping the extreme values unchanged. Next, for each stratum, the average value was estimated. The calculated mean square error between the numerical and experimental translated distributions indicated the material *cov* = 0.16 gave the best agreement.

It is worth reminding here that Koide et al. [7,8] measured in a laboratory the compressive strength *f_c_* only, whereas the uniaxial tensile strength *f_t_* (the generic mean value for random fields) in the initial case study was roughly estimated based on *f_c_* and the recommendations in Eurocode 2 [17]. Meanwhile, the stochastic results clearly showed that the uniaxial tensile strength *f_t_* needed correction. Hence, renewed SFEM analysis of beam LM200 was run with a modified uniaxial tensile strength *f_t_* = 3.48 MPa and with the material *cov* = 0.16. The total fracture energy *G_F_* and its ratio to the initial fracture energy *G_F_*/*G_f_* remained the same. This time, the obtained mean bending moment reached 704 Nm and was nearly the same as the experimental value of 702 Nm.

### 3.3. The Statistical Size Effect on the Beam Strength

**Figure 9:**
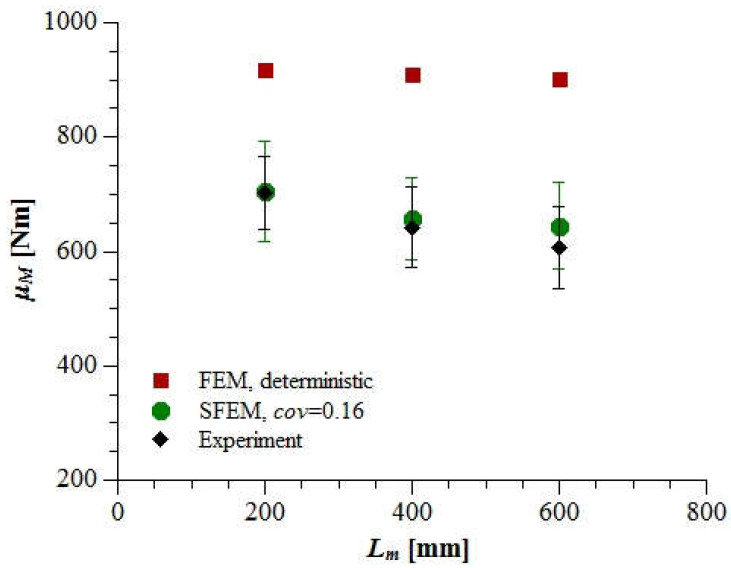
The mean ultimate bending moment (±standard deviation) of a beam with increasing span calculated based on SFEM simulations with *cov* = 0.16 compared with deterministic FEM analysis and the experimental results of Koide et al. (Copyright Permission, Proceedings of FRAMCOS-3, 1998 and Proceedings of ICASP-8, 2000) [7,8].

After calibrating the material and stochastic parameters based on the smallest beam (LM200), the remaining beams (LM400 and LM600) were simulated with the following mean values: *f_t_* = 3.48 MPa, *G_F_* = 80 N/m (*G_F_*/*G_f_* = 1.5) and *E_mod_* = 30 GPa, while the coefficient of variation was *cov* = 0.16 for all the material parameters independent of the beam size. For each beam size, 24 stochastic simulations were performed. The calculated mean ultimate bending moment *μ_M_* reached 658 Nm (LM400) and 645 Nm (LM600) and was 3 and 6% higher than the corresponding mean experimental value (Figure 9). The calculated standard deviation of *M* was similar in beams LM400 and LM600 and slightly higher in beam LM200 compared with the experimental values (Figure 9).

**Figure 10:**
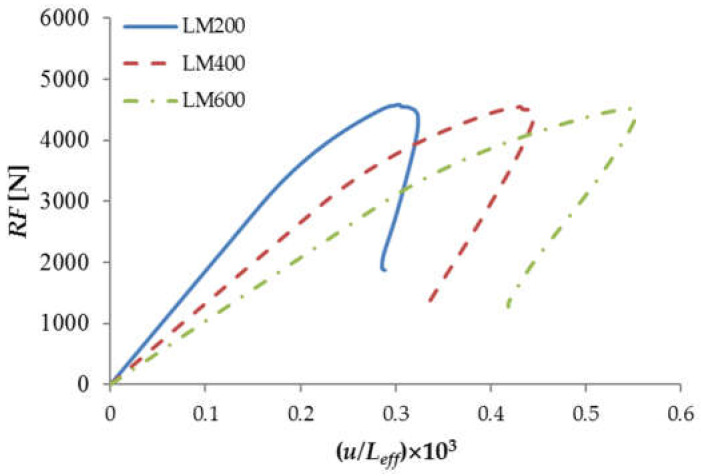
Normalized load-deflection diagrams from deterministic FEM simulations with various beam spans.

**Figure 11:**
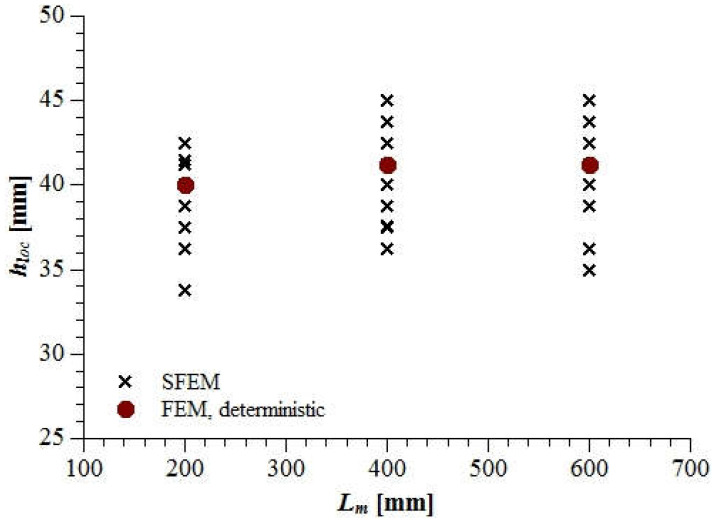
Measured localized zone height at the peak load from deterministic and stochastic FEM simulations with *cov* = 0.16.

Additional calculations were performed for the deterministic FEM models (with a constant and uniformly distributed *f_t_* = 3.48 MPa, *G_f_* = 52 N/m, *G_F_* = 80N/m and *E_mod_* = 30 GPa). Deterministic simulations confirmed the energetic size effect upon the beam’s nominal strength (in this case, the equivalent bending moment) was negligible when the beam depth *D* was constant. However, the deterministic effect of the varying beam span *L_eff_* was evident when comparing the beams’ brittleness. Figure 10 includes the normalized load-deflection diagrams illustrating that the longer beam, the more brittle the post-peak response (a so-called snap back behavior could be observed thanks to the applied arc-length method). When comparing the deterministic and stochastic results, the mean stochastic bending moment was 23, 28 and 29% lower than the corresponding deterministic value. The reduction was mainly caused by a locally weaker tensile strength inside the localized zone area (the width was always *w_loc_* = 6 mm, while the height *h_loc_* fluctuated around 41 m (Figure 11).

Generally, the agreement between SFEM results with *cov* = 0.16 and experimental outcomes (Figure 9) was satisfactory but not perfect. The increasing discrepancy between the mean values together with the beam size indicate that the simulated statistical size effect on the beam’s mean nominal strength should have been stronger. For this reason, SFEM analysis of all beams was performed once more with a higher material coefficient of variation *cov* = 0.24. The uniaxial tensile strength was *f_t_* = 4.2 MPa (calibrated again based on the mean bending moment *μ_M_* from 24 SFEM simulations of the smallest beam, LM200), whereas the other material parameters remained unchanged (*G_F_* = 80 N/m, *G_F_*/*G_f_* = 1.5 and *E_mod_* = 30 GPa). For each beam size, 24 SFEM simulations were executed.

**Figure 12:**
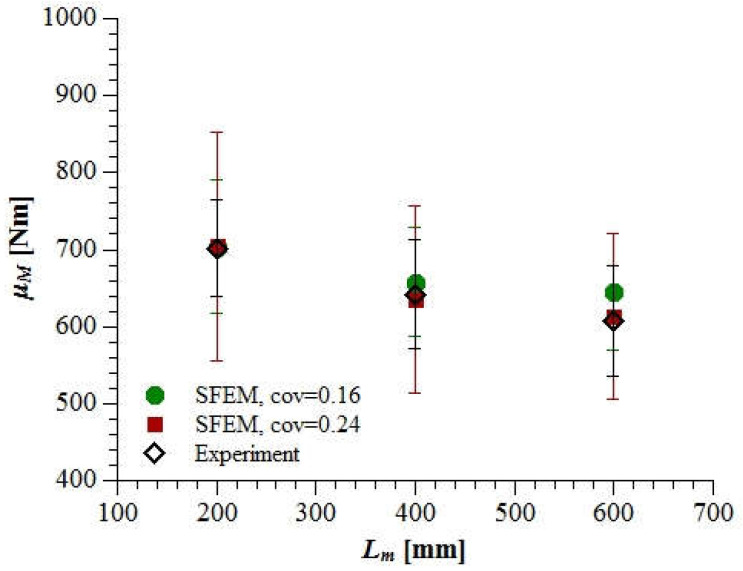
The mean ultimate bending moment (±standard deviation) of a beam with increasing span calculated based on SFEM simulations with *cov* = 0.16 and *cov* = 0.24 compared with the experimental results of Koide et al. (Copyright Permission, Proceedings of FRAMCOS-3, 1998 and Proceedings of ICASP-8, 2000) [7,8].

This time, the agreement between the laboratory measured and simulated mean ultimate bending moments for *cov* = 0.24 was nearly perfect (Figure 12). However, the scattering of results in the case of SFEM analysis with *cov* = 0.24 was significantly stronger than in the experiments. This may indicate the need to introduce the grafted Gaussian–Weibull probability distribution function for the concrete material properties in order to better reflect both the mean and the standard deviation of the results.

The effect of the simultaneously varying material properties was also examined. Referring to the results discussed above, where strong cross-correlation (*r* = 0.9) was adopted between *f_t_*, *G_f_* and *E_mod_*, the assumption of a constant *G_f_* and *E_mod_* increased the mean ultimate bending moment by 5% and decreased its standard deviation by 16% on average. On the other hand, when a linear dependence between *f_t_* and *G_f_* was applied (it was found that *E_mod_* affected only the initial beam stiffness), the resulting mean ultimate bending moment decreased by less than 2%, while its standard deviation was higher by 4% on average. These results demonstrate that the variation of the local uniaxial tensile strength *f_t_* was crucial for the statistical size effect.

## 4. Conclusions

The following conclusions can be drawn from the FEM investigation of plain concrete beams of varying lengths, summarizing both the deterministic and the stochastic approach with simultaneously and spatially varying *f_t_*, *G_f_* and *E_mod_* values:

- The spatial fluctuation of the local material properties was the source of the mean nominal strength reduction with an increasing beam length;
- The increasing material coefficient of variation *cov* contributed to the stronger statistical size effect on the mean nominal strength (i.e., the more pronounced reduction of the mean strength with the increasing beam length);
- The simulated statistical size effect with the material *cov* = 0.16, calibrated based on the bending moment variation of the shortest beam LM200, was too weak compared with the experimental results;
- The best approximation of the experimentally registered mean nominal strengths for increasing beam lengths was numerically obtained with the material *cov* = 0.24, but the calculated results’ variation was larger than that observed by Koide et al. [4,5];
- Deterministic analysis showed the increase in the beam length (with the constant depth) caused the more brittle post-peak behavior but did not change the nominal strength.

The future plan is to compare the presented results with similar SFEM analysis using the grafted Gaussian–Weibull cumulative probability distribution function.

## Data Availability

Not applicable.
